# Effectiveness of Tranexamic Acid in Reducing Hemorrhage in Isolated Blunt Solid Organ Injury

**DOI:** 10.7759/cureus.20473

**Published:** 2021-12-16

**Authors:** Vitaley Kovalev, Fanglong Dong, Sina Bagheri, David Wong, Matthew Wi

**Affiliations:** 1 General Surgery, Riverside University Health System Medical Center, Moreno Valley, USA; 2 General Surgery, Arrowhead Regional Medical Center, Colton, USA; 3 Emergency Medicine, Arrowhead Regional Medical Center, Colton, USA; 4 Internal Medicine, Western University of Health Sciences, Pomona, USA; 5 Surgery, Arrowhead Regional Medical Center, Colton, USA

**Keywords:** renal injury, blunt liver trauma, splenic trauma, liver injury, blunt organ injury, trauma, treanaximic acid (txa)

## Abstract

Introduction: There is considerable interest in the use of tranexamic acid (TXA) for the control of hemorrhages in trauma patients. Multiple recent studies found that TXA used in the setting of a suspected significant hemorrhage in trauma patients significantly reduced mortality. To date, there are no cited studies that specifically address hemorrhage due to solid organ injury (i.e., kidneys, liver, and spleen) and TXA use in humans. Our current research addresses whether TXA is effective in reducing complications and mortality from traumatic hemorrhage in the setting of a specific solid organ injury.

Methods: We conducted a retrospective observational cohort study utilizing propensity score matching at Arrowhead Regional Medical Center (ARMC) from February 1, 2009 to February 1, 2019. This study period marks five years prior to and five years after February 1, 2004, which is the date when TXA first started to be used at ARMC in the management of traumatic hemorrhage. We compared for statistical difference between corresponding injury types in the TXA and non-TXA groups.

Results: Before the propensity matching, there were 123 patients who received TXA and 118 patients who did not. After propensity match for age and injury severity score (ISS), 35 patients were included in each group. We found no statistically significant difference between TXA and non-TXA treatment groups in terms of mortality at 24 hours (p-value=0.4945), mortality at 48 hours (p-value=0.4945), and mortality at 28 days (p-value=0.7426). We found no statistically significant difference between the need for interventional radiology intervention at 72 hours (p-value=0.3932), surgical intervention at 72 hours (p-value=0.2123) and possible TXA related complications (p-value=1).

Conclusion: Although prior studies showed that TXA use in the setting of trauma may be beneficial, the specific candidate-selection criteria remain unclear. The results of our study suggest that the benefit from TXA in the setting of the isolated splenic, liver, and or renal injury may be negligible. We believe that this first-of-its-kind study adds to the growing body of knowledge about the utility of TXA and helps guide patient-selection criteria.

## Introduction

Trauma is a major cause of death and injury worldwide. An estimated 5.8 million people die every year because of traumatic injuries, and this comprises an estimated 10% of the world’s annual deaths [1]. Hemorrhage is one of the mechanisms by which death after trauma occurs. There is considerable interest in the use of tranexamic acid (TXA) for the control of hemorrhages in patients subjected to severe trauma.

The mechanism by which TXA helps control hemorrhage is by inhibiting the enzymatic breakdown of fibrin [2]. TXA does this by competitively inhibiting the activation of plasminogen to plasmin via binding to kringle domains [3]. The Clinical Randomization of an Antifibrinolytic in Significant Hemorrhage (CRASH)-2 trial published in 2010 was a landmark study that brought TXA to the forefront of discussion [4]. CRASH-2 trial found that TXA when administered within three hours of a traumatic event with suspected hemorrhage significantly reduced mortality. Subsequently, there has been some interest in identifying the utility of TXA in the setting of specific injuries. The CRASH-3 trial found that TXA decreased the risk of injury-related deaths in patients with mild-to-moderate head injury [5]. Similarly, a more recent meta-analysis found that the use of TXA for traumatic brain injury is associated with substantially reduced mortality and the extent of intracranial hemorrhage [6]. Our research of available literature however revealed no study which directly examined the utility of TXA for specifically isolated solid organ injuries (liver, kidney, and splenic injuries). We identified two papers that looked at the use of TXA for hemorrhage due to a liver injury in rat and porcine models. The first study looked at the effect of TXA-loaded trauma-targeted nanovesicles (T-tNVs), TXA-loaded control (untargeted) nanovesicles (TNVs), free TXA, and saline in a liver trauma model in rats [7]. When looking specifically at only the use of TXA, the study found that TXA provides significant improvement in the control of liver hemorrhage compared to the control group. A different paper looked at blood loss and coagulation after an induced hemorrhage following a blunt liver injury in the porcine model [8]. That study looked at whether human fibrinogen concentrate (FC) and prothrombin complex concentrate (PCC), administered as combined therapy with TXA, would provide additive effects for reducing blood loss. The study found that blood loss in all groups, including just the TXA group, was lower (P < 0.001) than in the control. No studies were found which looked specifically at solid organ injury hemorrhage (i.e., kidneys, liver, and spleen) and TXA use in humans. Our study seeks to fill this gap in research in hopes of optimizing the clinical decision-making process when it comes to the use of TXA as applied to specific blunt organ injuries.

Our research question is whether TXA is effective in reducing complications and mortality from traumatic hemorrhage in the setting of a specific solid organ injury. The purpose of the study is to elucidate the utility of TXA with respect to specific traumatic organ injuries. Our hypothesis is that TXA will significantly reduce the risk of hemorrhage in blunt solid organ injury.

## Materials and methods

This retrospective observational cohort study was conducted at Arrowhead Regional Medical Center (ARMC), a level I trauma center, from February 1, 2009 to February 1, 2019, five years prior to and five years after February 1, 2004, which is the date when ARMC began first using TXA in the management of traumatic hemorrhage. Out inclusion criteria were all trauma patients with blunt liver, spleen, and renal injuries who were seen at ARMC within the above-mentioned time frame. Our exclusion criteria were patients who sustained an isolated vascular injury, penetrating trauma, patients who did not sustain a liver, spleen, or renal injury, and patients who were deceased upon arrival to the emergency department. All patients older than 18 years of age who sustained traumatic injuries with signs of hemorrhagic shock were considered for TXA treatment. Patient selection in the prehospital setting was determined by emergency medical services in the field. TXA was given in two doses following the same protocol used in the CRASH-2 trial [4]. The first dose was 1 gram of TXA in 100 mL of 0.9% normal saline infused over 10 minutes. Upon arrival at ARMC, patients who received prehospital TXA were re-assessed by the trauma team. Those who continued to meet the study criteria received a second dose of 1 gram of TXA infused over eight hours. Patients were included in the TXA group who received at least one dose of TXA. Injury severity scores (ISS) of all patients were double-checked and corrected if needed. The control group patients were matched to the TXA group patients through propensity scoring based on age and ISS. We also recorded whether the Glasgow Coma Scale (GCS) of the patient upon presentation was less than 8 and whether the initial systolic blood pressure (SBP) upon presentation was less than 90. The primary outcome was mortality measured at 24 hours, 48 hours, and 28 days. Additional variables included were the need for interventional radiology (IR) intervention for hemorrhage control within 72 hours of admission and the need for surgical intervention to control hemorrhage within 72 hours of admission. Patients who underwent surgical intervention within 72 hours of admission for non-hemorrhage-related indications, such as repair of orthopedic injuries and repair of hollow viscus injury, were excluded from the “surgical intervention” group. A large systematic review by Ker et al. found that TXA was not associated with an increased incidence of prothrombotic complications [9]. However, since prothrombotic complications are still a concern of TXA administration, we included the incidence of deep vein thrombosis (DVT), pulmonary embolism (PE), myocardial infarction (MI), cerebrovascular accident (CVA) in our study population.

Specific injuries were categorized by their severity (radiological or intra-operative grading), by specific organ injured, and whether TXA was used. We compared for statistical difference between corresponding injury types between TXA and non-TXA groups. All statistical analyses were conducted using the SAS software for Windows version 9.4 (Cary, North Carolina, USA) and R. To eliminate the effect of confounders (age and ISS) on the primary outcomes (mortality at 24 hours, 48 hours, and 28 days, need for IR intervention at 72 hours and need for surgical intervention at 72 hours), an exact propensity matching was conducted using the R-package MatchIt. Descriptive statistics were presented as means and standard deviations for continuous variables, along with frequencies and proportions for categorical variables. Chi-square tests were conducted to assess the association between categorical variables and TXA administration status. Fisher Exact test was used for variable values less than five. Independent T-tests were conducted to compare the continuous variables between the TXA administration status. All statistical tests were two-sided. P-value < 0.05 was considered statistically significant.

## Results

Before the propensity matching, there were 123 patients who received TXA and 118 patients who did not receive TXA. After the propensity match based on age and ISS, 35 patients were included in each group. As expected by the propensity matching, there was no statistically significant difference based on age (p-value=0.9726), and ISS (p-value=0.9905) (Table 1). Additionally, we found no statistically significant difference based on gender (p-value=0.7708), the number of patients who had an initial SBP less than 90 mmHg (p-value=0.1638), and patients who presented with an initial GCS of less than 8 (p-value=0.4030). There was no statistically significant difference in the distribution of grades of liver, renal and splenic injuries between the TXA and non-TXA groups. Regarding the outcome variables, we found no statistically significant difference between the TXA and non-TXA groups in terms of mortality at 24 hours (p-value=0.4945), mortality at 48 hours (p-value=0.4945), and mortality at 28 days (p-value=0.7426) (Table 2). Additionally, we found no statistically significant difference between the IR intervention at 72 hours (p-value=0.3932), surgical intervention at 72 hours (p-value=0.2123) and complications (p-value=1).

**Table 1:** Comparison of baseline characteristics between the TXA and the non-TXA groups. ISS - injury severity score, SBP - systolic blood pressure, GCS - Glasgow Coma Scale

	Non TXA (n=35)	TXA (n=35)	P-value
Gender			0.7708
Female	7 (20%)	8 (22.9%)	
Male	28 (80%)	27 (77.1%)	
Age	32.71 ± 13.91	32.83 ± 13.84	0.9726
ISS	24 ± 8.63	24.26 ± 8.52	0.9005
Initial SBP < 90 mmHg	1 (3%)	4 (11%)	0.1638
Initial GCS < 8	7 (20%)	10 (29%)	0.4030
Renal Injury Grade			
No injury	29 (83%)	30 (86%)	1
I	1 (3%)	3 (9%)	0.6151
II	2 (6%)	0 (0%)	0.4933
III	1 (3%)	2 (6%)	1
IV	1 (3%)	0 (0%)	1
V	1 (3%)	0 (0%)	1
Spleen Injury Grade			
No injury	20 (57%)	22 (63%)	0.847
I	3 (9%)	2 (6%)	1
II	9 (26%)	5 (14%)	0.2436
III	1 (3%)	3 (9%)	0.6142
IV	2 (6%)	3 (9%)	1
V	0 (0%)	0 (0%)	1
Liver Injury Grade			
No injury	21 (60%)	20 (57%)	1
I	4 (11%)	6 (17%)	0.7343
II	6 (17%)	6 (17%)	1
III	1 (3%)	2 (6%)	1
IV	1 (3%)	1 (3%)	1
V	2 (6%)	0 (0%)	0.4928

**Table 2:** Comparison of outcome variables between the TXA and the non-TXA groups. IR - interventional radiology, DVT - deep vein thrombosis, PE - pulmonary embolism, MI - myocardial infarction, CVA - cerebrovascular accident

	Non TXA (n=35)	TXA (n=35)	P-value
Mortality at 24 hours			0.4945
Alive	29 (82.9%)	31 (88.6%)	
Dead	6 (17.1%)	4 (11.4%)	
Mortality at 48 hours			0.4945
Alive	29 (82.9%)	31 (88.6%)	
Dead	6 (17.1%)	4 (11.4%)	
Mortality at 28 days			0.7426
Alive	29 (82.9%)	30 (85.7%)	
Dead	6 (17.1%)	5 (14.3%)	
IR intervention at 72 hours	2 (5.7%)	4 (11.4%)	0.3932
Surgical intervention at 72 hours	10 (28.5%)	15 (42.8%)	0.2123
Complications (DVT, PE, MI, CVA)	0	0	1

## Discussion

This retrospective observational study with propensity score-matched cohorts investigated the use of prehospital TXA in the setting of suspected solid organ traumatic injuries. This is the first study in human subjects, which looked at the effect of TXA administration specifically in the setting of blunt solid organ injury (kidney, liver, spleen). Because this is a retrospective study and randomization was not possible, we employed propensity-score matching by age and ISS to eliminate baseline differences between the TXA and non-TXA groups. This matching resulted in statistically similar groups across unmatched variables as well, including the incidence of GCS scores less than 3, the incidence of initial SBPs less than 90 mmHg, and patterns of injury. We found that TXA administration did not decrease the mortality at 24 hours, 48 hours, and 28 days. We also found that the TXA administration group did not reduce the need for IR intervention for hemorrhage control, nor surgical intervention for hemorrhage control at 72 hours. Finally, there were no reported potentially TXA-related complications in either cohort. Although we had a relatively small cohort, this finding is consistent with prior studies that have not shown an increase in prothrombotic complications after TXA administration [9].

Prior studies reported a reduction in the 28-day mortality rate after TXA administration. CRASH-2 trial reported an all-cause mortality reduction to 14.5% in the TXA group compared to 16.0% in the non-TXA group at 28 days (p-value = 0.0035) [4] and Neeki et al. reported mortality reduction at 3.6% in the TXA group vs 8.3% for the non-TXA group, also only at 28 days [10]. Our study differs in that we specifically looked only at patients with solid organ injuries and excluded patients who may have sustained hemorrhage from other causes. Because prior studies in porcine and rat models did find a reduction in hemorrhage specifically in blunt liver injury after TXA administration, we expected to see a similar benefit in the setting of acute trauma in humans [7,8]. Given that the effect of TXA on mortality in prior studies was relatively small combined with the highly restrictive inclusion criteria of our study, we suspect that our study may have been underpowered. Another foreseen limitation is how these results ought to be put into practice. Current protocols in the setting of trauma suggest that TXA administration be used in the out-of-hospital setting prior to any knowledge of the specific injuries the patient has sustained [4]. Therefore, the benefit of TXA in a specific injury pattern in the out-of-hospital setting may not be relevant. TXA has also been widely investigated in the in-patient perioperative settings and there is evidence that TXA reduces perioperative bleeding in coronary artery surgery, orthopedic surgery, and peripartum hemorrhage [11-13]. We believe that the results of this study can be helpful in the specific situation where a solid organ injury is identified on appropriate imaging in a trauma patient after arrival to the emergency department and the patient displays evidence of uncompensated hemorrhage. Another limitation of this study is patient selection. The researchers tried to select patients with isolated solid organ injury; however, injuries rarely occur in an isolated fashion and most injury patterns are a mix of vascular, soft tissue, solid organ, skeletal and other injuries. As mentioned above, we made an effort to exclude patients with non-solid-organ sources of major hemorrhage by excluding patients with major vascular injury. Lastly, our study also did not use coagulation testing, such as thromboelastography (TEG) or rotational thromboelastometry (ROTEM) prior to administration of TXA. Previous studies are inconsistent on whether monitoring coagulation testing prior to administration of TXA would provide any clinically significant information [14,15].

## Conclusions

Prior studies showed that TXA use in the setting of trauma may be beneficial; however, the specific candidate-selection criteria remain unclear. The results of our study suggest that the benefit from TXA in the setting of the isolated splenic, liver, and/or renal injury may be negligible. We believe that this first-of-its-kind study adds to the growing body of knowledge about the utility of TXA and helps guide patient selection criteria.
